# Multi-OMICs analysis reveals metabolic and epigenetic changes associated with macrophage polarization

**DOI:** 10.1016/j.jbc.2022.102418

**Published:** 2022-08-27

**Authors:** Mark L. Sowers, Hui Tang, Vipul K. Singh, Arshad Khan, Abhishek Mishra, Blanca I. Restrepo, Chinnaswamy Jagannath, Kangling Zhang

**Affiliations:** 1 Department of Pharmacology and Toxicology, University of Texas Medical Branch, Galveston, Texas, USA; 2 Department of Pathology and Genomic Medicine, Center for Molecular and Translational Human Infectious Diseases Research Houston Methodist Research Institute, Weill-Cornell Medicine, Houston, Texas, USA; 3 University of Texas Health Science Center at Houston, School of Public Health, Brownsville, Texas, USA

**Keywords:** Macrophage, macrophage polarization, multiomics, histone modifications, epigenetics, metabolism and epigenetics, mass spectrometry., 5-methylTHF, 5-methyltetrahydrofolate, ETC, electron transport chain, GM-CSF, granulocyte-macrophage colony stimulating factor, iNOS, inducible nitric oxide synthase, MS, mass spectrometry, NAA, N-acetyl-aspartic acid, NAG, N-acetyl-glutamate, NAO, N-acetyl-ornithine, NOS, nitric oxide synthase, OAA, oxaloacetate, O-BHA, O-benzylhydroxylamine, OxPhos, oxidative phosphorylation, PBMC, peripheral blood mononuclear cell, ROS, reactive oxygen species, SAM, S-adenosylmethionine, TCA, tricarboxylic acid, TMT, tandem mass tag

## Abstract

Macrophages (MФ) are an essential immune cell for defense and repair that travel to different tissues and adapt based on local stimuli. A critical factor that may govern their polarization is the crosstalk between metabolism and epigenetics. However, simultaneous measurements of metabolites, epigenetics, and proteins (phenotype) have been a major technical challenge. To address this, we have developed a novel triomics approach using mass spectrometry to comprehensively analyze metabolites, proteins, and histone modifications in a single sample. To demonstrate this technique, we investigated the metabolic-epigenetic-phenotype axis following polarization of human blood–derived monocytes into either ‘proinflammatory M1-’ or ‘anti-inflammatory M2-’ MФs. We report here a complex relationship between arginine, tryptophan, glucose, and the citric acid cycle metabolism, protein and histone post-translational modifications, and human macrophage polarization that was previously not described. Surprisingly, M1-MФs had globally reduced histone acetylation levels but high levels of acetylated amino acids. This suggests acetyl-CoA was diverted, in part, toward acetylated amino acids. Consistent with this, stable isotope tracing of glucose revealed reduced usage of acetyl-CoA for histone acetylation in M1-MФs. Furthermore, isotope tracing also revealed MФs uncoupled glycolysis from the tricarboxylic acid cycle, as evidenced by poor isotope enrichment of succinate. M2-MФs had high levels of kynurenine and serotonin, which are reported to have immune-suppressive effects. Kynurenine is upstream of *de novo* NAD^+^ metabolism that is a necessary cofactor for Sirtuin-type histone deacetylases. Taken together, we demonstrate a complex interplay between metabolism and epigenetics that may ultimately influence cell phenotype.

Macrophages (MФs) are essential phagocytes that mediate antimicrobial immunity. MФ precursors, monocytes, enter circulation and home to tissues where they differentiate into tissue-specific MФs that express distinct markers. Multiple phenotypes have been described that perform site-specific functions depending on the tissue environment (1, 2, 3). MФ differentiation and activation is a complex process involving orchestrated signaling induced by cytokines, chemokines, and interactions with pathogen-associated molecular patterns and damage-associated molecular patterns (4). Classically, activated MФs are ‘proinflammatory’ and referred to as M1-MФs, whereas alternatively activated MФs are ‘anti-inflammatory’ and referred to as M2-MФs (5). M1-MФs can be induced by IFN-γ from monocytes or resting MФs (M0-MФ), and M2-MФs are induced by IL-4, IL-10, and IL-13 (6). In addition, multiple subsets of M2-MФs (M2a, M2b, and M2c) have also been described (7).

MФ polarization helps the immune system balance between the need to kill pathogens (M1-MФs) and to perform repair following infection- and cytokine-related damage (M2-MФs) (8). Improper MФ polarization can contribute to the pathophysiology of metabolic syndrome (9, 10, 11), infectious and autoimmune diseases (12), asthma (13), and cancer (10, 14). A better understanding of MФ polarization may allow us to develop therapeutic strategies for tuning polarization.

Recent studies have shed some light on the interplay that exists between epigenetics and metabolism that may be involved or even govern macrophage polarization (15, 16). Previous studies, primarily using mouse MФs, show a striking contrast between transcriptional and metabolic profiles between M1-MФs and M2-MФs (10, 17, 18, 19, 20). Typically inducible nitric oxide synthase (iNOS) is reported to be upregulated in M1-MФs, resulting in arginine catabolism to citrulline and nitric oxide (NO), as well as produce reactive oxygen and nitrogen species (21). In contrast, M2-MФs instead convert arginine into ornithine and urea using ARG1. Furthermore, M1-MФs are characterized by enhanced glycolysis, an upregulated pentose phosphate pathway, and decreased oxidative phosphorylation (OxPhos) while M2-MФs are characterized by largely the opposite (11, 19, 22). Furthermore, metabolites and nutrients in the microenvironment, including amino acids, glucose, fatty acids, hormones, vitamins, and even oxygen, can further influence polarization (23, 24, 25, 26, 27, 28, 29).

Histone epigenetic regulation can directly influence gene expression. Enzymes that add or remove these modifiers to chromatin alter the physical accessibility of a set of genes and their promoters (30, 31). Both mathematical models and experimental observations confirm acetylation changes occur depending upon the metabolic state. For example, a shift from low to high glucose significantly affects histone acetylation levels, as do changes in acetate, hypoxia, or starvation of certain amino acids (32, 33, 34, 35, 36, 37). Histone acetylation and methylation are dependent upon their required acetyltransferase and methyltransferase cofactors, acetyl-CoA and S-adenosylmethionine (SAM), respectively. Acetyl-CoA is derived from pyruvate, the final product of glycolysis, as well as β-oxidation of fatty acids. SAM is formed from methionine, which can be imported or synthesized from homocysteine and 5-methyltetrahydrofolate (5-methylTHF), which requires B_12_ and folate. Serine or glucose-derived serine, is the source of carbon for 5-methylTHF. Consequently, epigenetic markers are a direct consequence of multiple overlapping metabolic pathways that are differentially activated in polarized macrophages.

While epigenetic regulation is closely tied to the synthesis and availability of the necessary cofactors, other factors produced by macrophages or in the microenvironment can further influence epigenetics. As an example, vitamin B_12_ can be irreversibly bound to NO, which is upregulated in M1-MФs and inhibits *de novo* methionine synthesis (38, 39, 40, 41). In addition, others have demonstrated that itaconic acid (19, 42, 43) can inhibit B_12_-dependent enzymes, and hypoxia can block intracellular uptake of B_12_ (44). In addition, others have demonstrated an accumulation of 5-methylTHF in M2-MФs relative to M1-MФs (45).

Previous approaches to measure metabolites, epigenetics, and protein expression (phenotype) have required multiple techniques and a variety of instruments including one or more kinds of mass spectrometers, Western blot, quantitative PCR, and other techniques. Each of these approaches is specialized and thus can limit the study of the metabolic-epigenetic-phenotype axis. Herein, we describe a novel triomics method to simultaneously analyze metabolites, histone modifications (epigenetics), and protein expression (phenotype) using MФs polarized *in vitro*. Secondly, we describe a mass spectrometric method to trace glucose incorporation into histone modifications (acetylation) that is based on our previous report of tracing histone methylation derived from serine (46). Our work described herein provides an analytical platform to study the complex crosstalk between metabolism, epigenetics, and cell plasticity. We demonstrate this by applying our approach to study MФ polarization

## Results

### Triomics analysis of metabolites, proteins, and histone modifications

**Figure 1:**
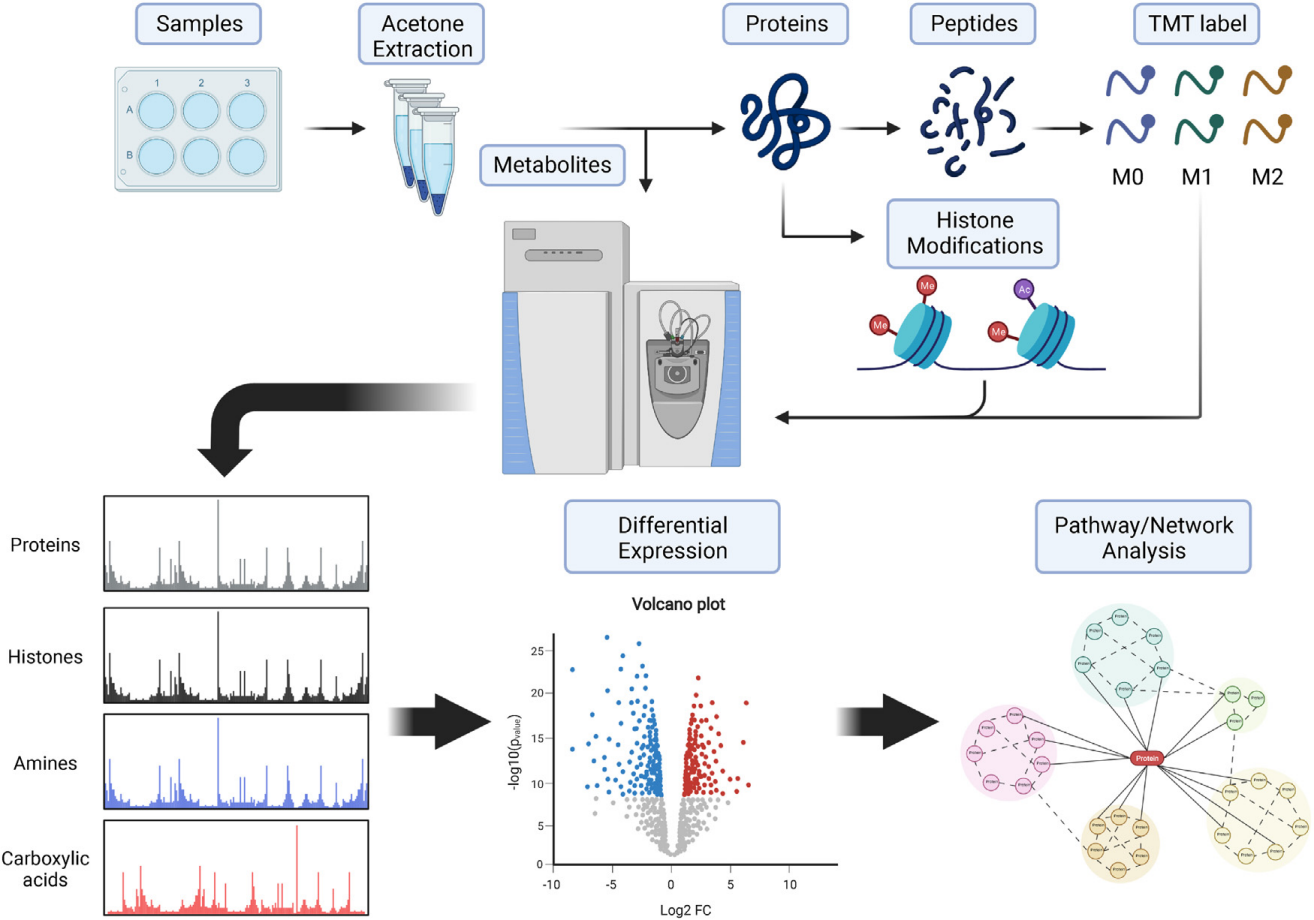
**Workflow for triomics for analysis of metabolites, histone modifications (epigenetics), and protein expression (phenotype).** Cell lysates of M0-, M1-, and M2-macrophages (MФs) are immersed into acetone for extraction of metabolites (supernatants) and proteins (precipitates). A portion of metabolite extract is derivatized by dansylation to analyze primary amines and another portion is derivatized by O-BHA to analyze carboxylic acids by nano-LC-MS/MS. Proteins are resolved by SDS-PAGE, and gel bands containing histones (MW < 20K) are cut for in-gel digestion and histone modifications are analyzed by nano-LC-MS/MS using parallel reaction monitoring. Proteins are in-solution digested by trypsin/Lys-C. Peptides are labeled by TMT6plex. TMT6-labeled peptides are analyzed by nano-LC-MS/MS to determine protein expression changed and perturbed protein pathways. MW, molecular weight; O-BHA, O-benzylhydroxylamine; TMT, tandem mass tag.

**Figure 2:**
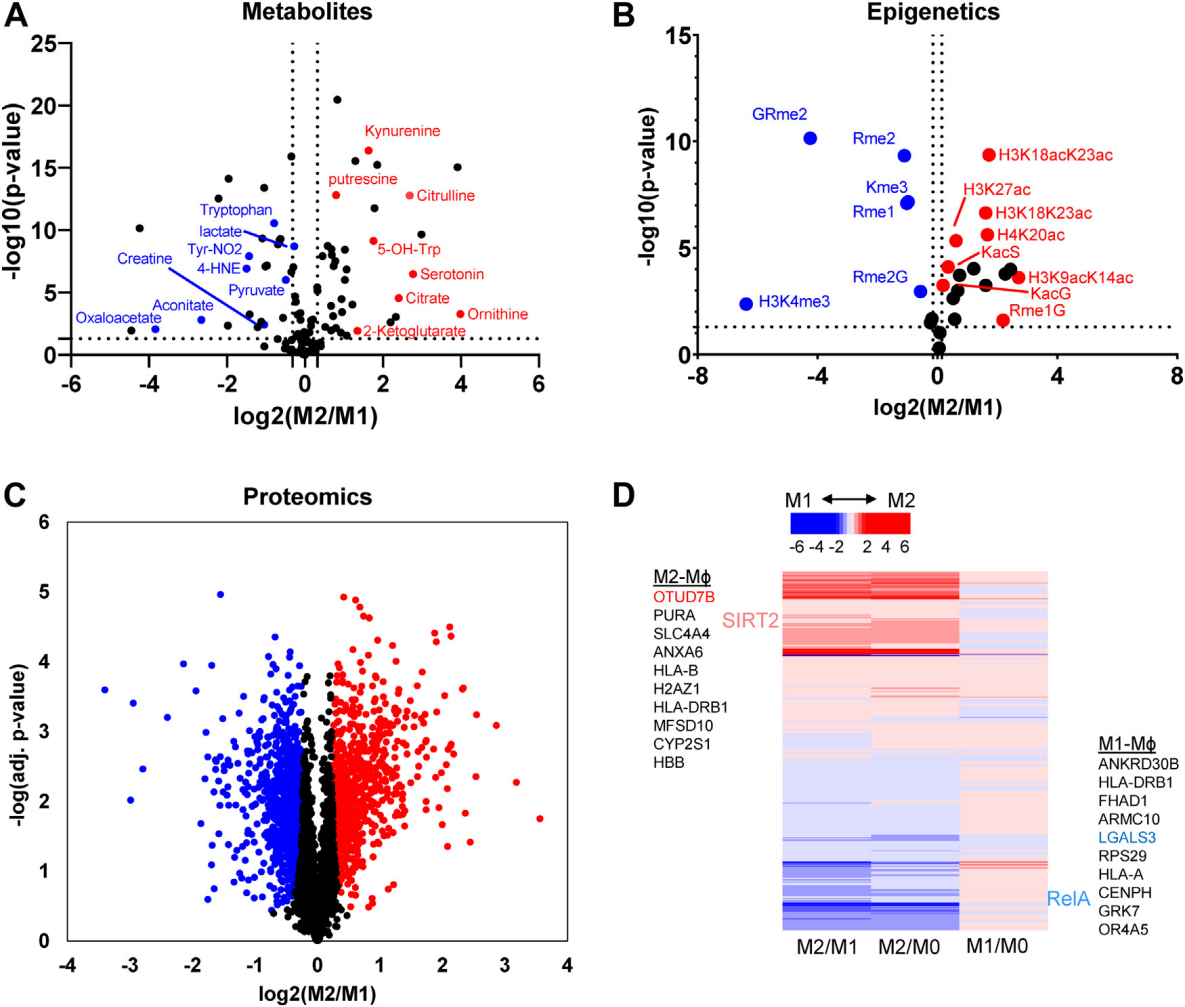
**Alteration of metabolites, histone acetylation and methylation, and protein expression in M1- and M2-Mɸs.***A*, volcano plot of the fold change in metabolites of M1- *versus* M2-MФs. The *x*-axis is the log_2_ of fold changes and the *y*-axis is the significance of the change of each corresponding metabolite with log_10_ (*p*-value) calculated by Student's two-way *t* test from at least eight consecutive LC-MS/MS runs from pooled biological samples. Two separate sample preparations were prepared from three wells of 6-well plate. *B*, volcano plot of the fold change in histone acetylation and methylation in M1- *versus* M2-MФs. The plot also includes methylation of arginine, lysine amino acids or two amino acid peptides. Data were acquired from the same sample preparation as in (*A*). Biological and technical replicates and statistical analyses are also the same as in (*A*). *C*, volcano plot of the fold change of proteins in M1- *versus* M2-MФs. Protein quantification was performed with one of the two sample preparations and one additional technical replicate was applied by labeling each sample (M0-, M1-, and M2-MФs) with two TMT tags. Significance of fold changes was done with the Perseus software based on FDR set to 0.01 (1%) and s0 set to 0.32 (∼1.25× fold change cutoff). *D*, heat map analysis of upregulated and downregulated proteins in M2-MФ compared to M1-MФ and both relative to M0-MФ. FDR, false discovery rate; TMT, tandem mass tag.

Figure 1 outlines our triomics workflow where we extract proteins, metabolites, and histones from single samples. After isolating and quantifying metabolites, we can visualize differential expression with a volcano plot comparing M1- and M2-MФs (Fig. 2*A*). A total of 112 metabolites were identified and quantified. The authenticity of metabolites was determined based on the accuracy of the precursor ions, which was set at a threshold of 5.0 ppm. The majority were further validated by MS2 fragment ions that corresponded to their chemical structures.

A total of 15 modified histone peptides were quantified (Fig. S1). Figure 2*B* shows a volcano plot displaying the fold changes of modifications in M2-MФs *versus* M1-MФs with significance (*p*-value) of change calculated from up to eight measurements of a pooled sample containing three biological samples. The data demonstrate that acetylation of histone H3 and H4 was consistently higher in M2-MФs than in M1-MФs. Additionally, dipeptide acetylation of KS and KG, detected in our metabolomics assay, was higher in M2-MФs than in M1-MФs. This suggests that these acetylated dipeptides could be degradation products of acetylated proteins (Fig. 2*A*).

As for methylated histone modifications, H3K4me3 was significantly higher in M1-MФs than M2-MФs. Methylation at other sites including H3K9 (monomethylation, dimethylation, and trimethylation), H3K27 trimethylation, H3K36 monomethylation, K79 monomethylation, and H4K20 monomethylation, dimethylation, and trimethylation was either lower in M1-MФs compared to M2-MФs or unchanged between them (Figs. 2*B* and S1). This suggests that there are significant alterations to the histone epigenetic code between M1- and M2-MФs, with histone acetylation broadly being elevated in M2-MФs and methylation being downregulated or unchanged.

A total of 2937 proteins were quantified with statistical significance (q-value ≤ 0.05). Figure 2*C* is a volcano plot showing the relationship of log_2_ fold change of proteins differentially expressed in M2-MФs *versus* M1-MФs and their statistical significance. About 866 proteins were downregulated (*blue*) and 710 proteins were upregulated (*red*) in M2-MФs *versus* M1-Mφs by >1.25 fold. Figure 2*D* illustrates a heat map of protein expression in M1- and M2-MФs compared to M0-MФs and between M1- and M2-MФs. Among the 10 most upregulated proteins, LGALS3/Galectin 3 was elevated in M1-MФs, consistent with our previous RNA-seq analysis (47). LGALS3 is associated with autophagy-dependent antibacterial activity (48). Also, consistent with our transcriptome analysis, RelA was significantly upregulated in M1-MФs, whereas SIRT2 was upregulated in M2-MФs. SIRT2 is known to regulate the expression of RelA deacetylation, thereby modulating its function (49).

**Figure 3:**
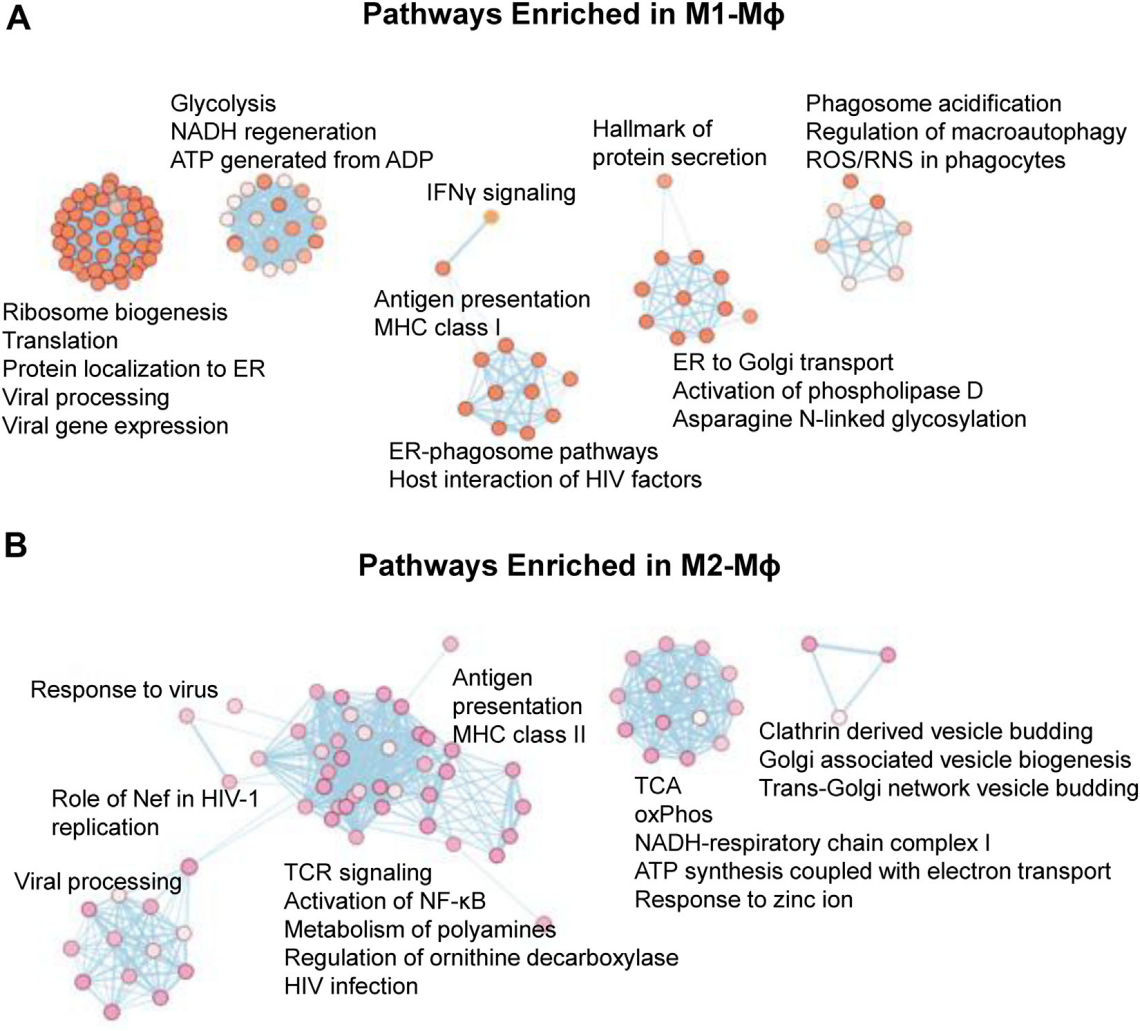
**Enriched protein pathways in M1-Mɸ and M2-Mɸ.***A*, enriched pathways built from upregulated proteins in M1-MФs. *B*, enriched pathways built from upregulated proteins in M2-MФs.

We then took the differentially expressed proteins for pathway enrichment analysis, using g:Profiler and Cytoscape with EnrichmentMap for visualization (50). IFN-γ signaling, response to type I interferon, NADH-respiratory chain complex 1, MHC-I–dependent antigen processing, glycolysis, and reactive oxygen species (ROS)/nitric oxide synthase (NOS) in phagocytes were among the enriched pathways in M1-MФs (Fig. 3*A*). In comparison, antigen presentation MHC class II, tricarboxylic acid (TCA) cycle, and OxPhos were enriched in M2-MФs (Fig. 3*B*).

### Arginine metabolism and arginine methylation

**Figure 4:**
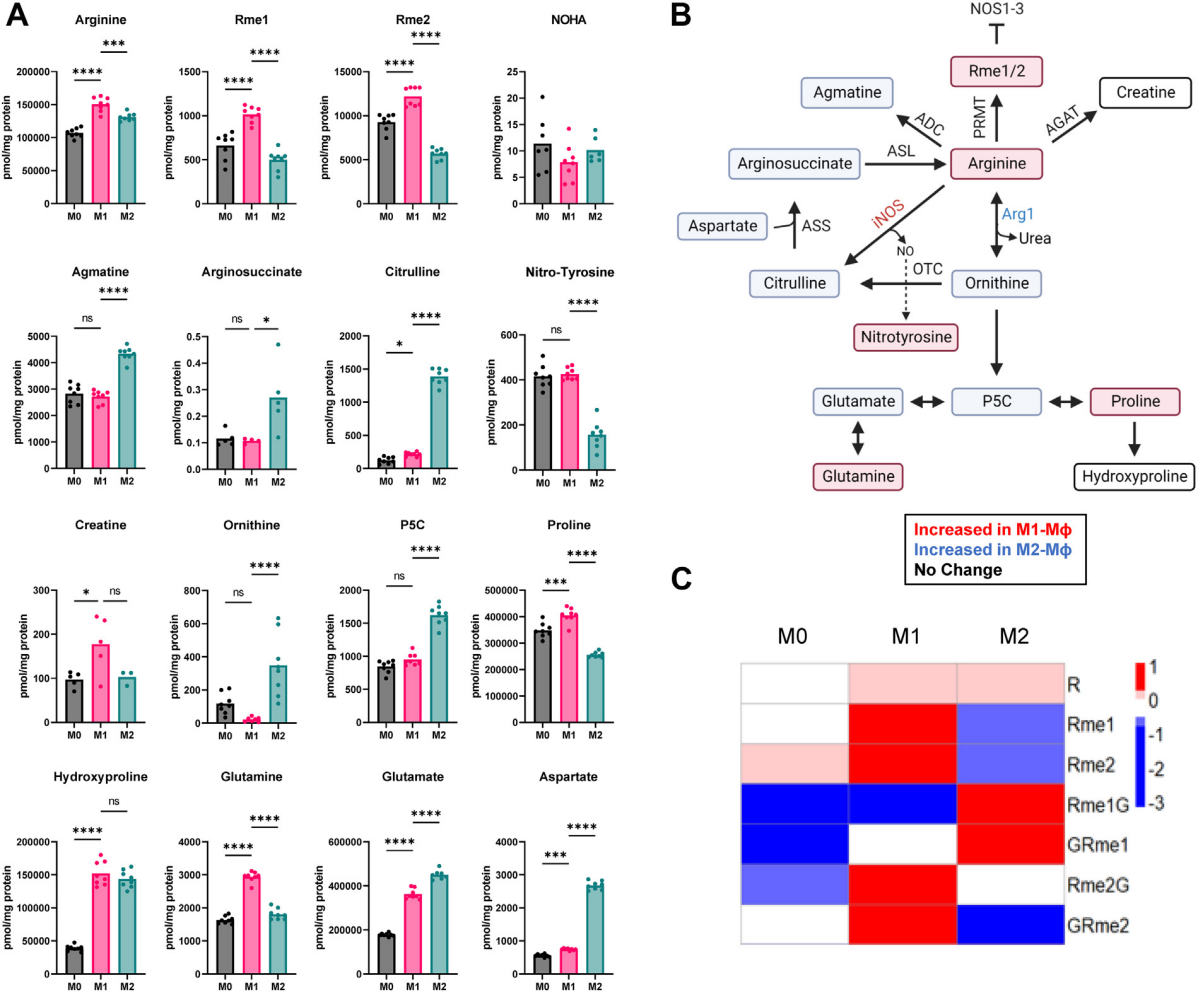
**Arginine metabolism and methylation in M1-Mɸ *versus* M2-Mɸ.***A*, concentrations of arginine pathway metabolites in M0-, M1-, and M2-MФs measured by LC-MS. Concentrations were calculated by normalizing the signal intensity relative to an internal standard of known concentration, 3,3,2-^2^H-serine. *B*, arginine pathway showing metabolites framed in *red* or *green* colors indicating an increase in either M1-MФ or M2-MФ. PMRT, protein arginine methyltransferase; ADC, arginine decarboxylase; AGAT, arginine:glycine amidinotransferase; ASL, argininosuccinate lyase; ASS, argininosuccinate synthase; OAT, ornithine aminotransferase; OTC, ornithine transcarbamylase. *C*, heat map showing differential levels of arginine methylation in M0-, M1-, and M2-MФs.

iNOS is a hallmark in activated M1-MФs. iNOS consumes arginine and metabolizes it to citrulline, producing NO. On the other hand, M2-MФs are frequently defined by the high expression of ARG1, which instead metabolizes arginine into ornithine and urea. While ornithine was elevated in M2-MФs, we did not see higher citrulline in M1-MФs as expected. Instead, both citrulline and ornithine were significantly higher in M2-MФs (Fig. 4, *A* and *B*). Despite this, we found evidence of NO oxidation in M1-MФs. We detected the NOS intermediate, N^G^-hydroxy-arginine (NOHA), although it was not significantly different between different MФs. However, NO can also react with tyrosine to form nitrotyrosine, which was found significantly higher in M1-MФs compared to M2-MФs (Fig. 4*A*). The high levels of citrulline in M2-MФs, rather than in the M1-MФs, are best explained by ornithine conversion into citrulline by ornithine transcarbamylase. Ornithine can then be metabolized to pyrroline 5-carboxylate, which can be further metabolized into proline or glutamate (Fig. 4, *A* and *B*). Overall, we find that M2-MФs elevate ornithine and citrulline levels likely due to conversion of arginine to ornithine and ornithine to citrulline, by ARG1 and ornithine transcarbamylase, respectively.

Alternatively, arginine can instead be metabolized to creatine or agmatine. Agmatine was higher in M2-MФs. Agmatine is a precursor of polyamines, which can suppress iNOS expression after inflammation, potentially playing an anti-inflammatory role (51, 52). Alternatively, arginine can be combined with glycine to produce ornithine and guanidinoacetate. Guanidinoacetate can then be converted to creatine following methylation using SAM. However, we found creatine levels in M2-MФs were not statistically different while absolute levels of agmatine were 10× to 20× higher than creatine. This suggests arginine is preferentially converted to agmatine.

Additionally, arginine residues in proteins can be methylated by arginine methyltransferases (PRMTs). These proteins can be catabolized to produce monomethylated and dimethylated arginine (Rme1 & Rme2) as well as dipeptides, GRme2 and Rme2G. These were all higher in M1-MФs compared to M2-MФs (Fig. 4*C*). Based on mass spectrometry (MS), arginine dimethylation was predominately asymmetric because the MH^+^-45 ion (loss of dimethylamine) was detected but the symmetric demethylation signature MH^+^-31 ion (loss of methylamine) was not (53). Interestingly monomethylated arginine, also known as N-mono-methyl-L-arginine, and asymmetric dimethylated arginine are known to modulate NO production by competing with arginine for NOS binding (54).

In contrast, monomethylated arginine dipeptides, Rme1G and GRme1, were significantly lower in M1-MФs. We were unable to detect PRMT1, which is the major asymmetric arginine dimethyltransferase (55, 56). Protein arginine methyltransferase 5 (PRMT5) was the only arginine methyltransferase among PRMT1-8 detected by proteomics and was upregulated in M2-MФs. PRMT5 can methylate histone H4R3 via monomethylation and symmetric dimethylation to repress gene expression. In addition, previous studies show that PRMT5 can methylate RelA-R30 to regulate the transcription of a subset of TNF-α–induced proinflammatory genes, including CXCL10 (57, 58). This intriguing data from our metabolic analysis warrants additional studies on the role of arginine metabolism and methylation, especially in the context of MФ polarization and activation.

### Tryptophan metabolism

**Figure 5:**
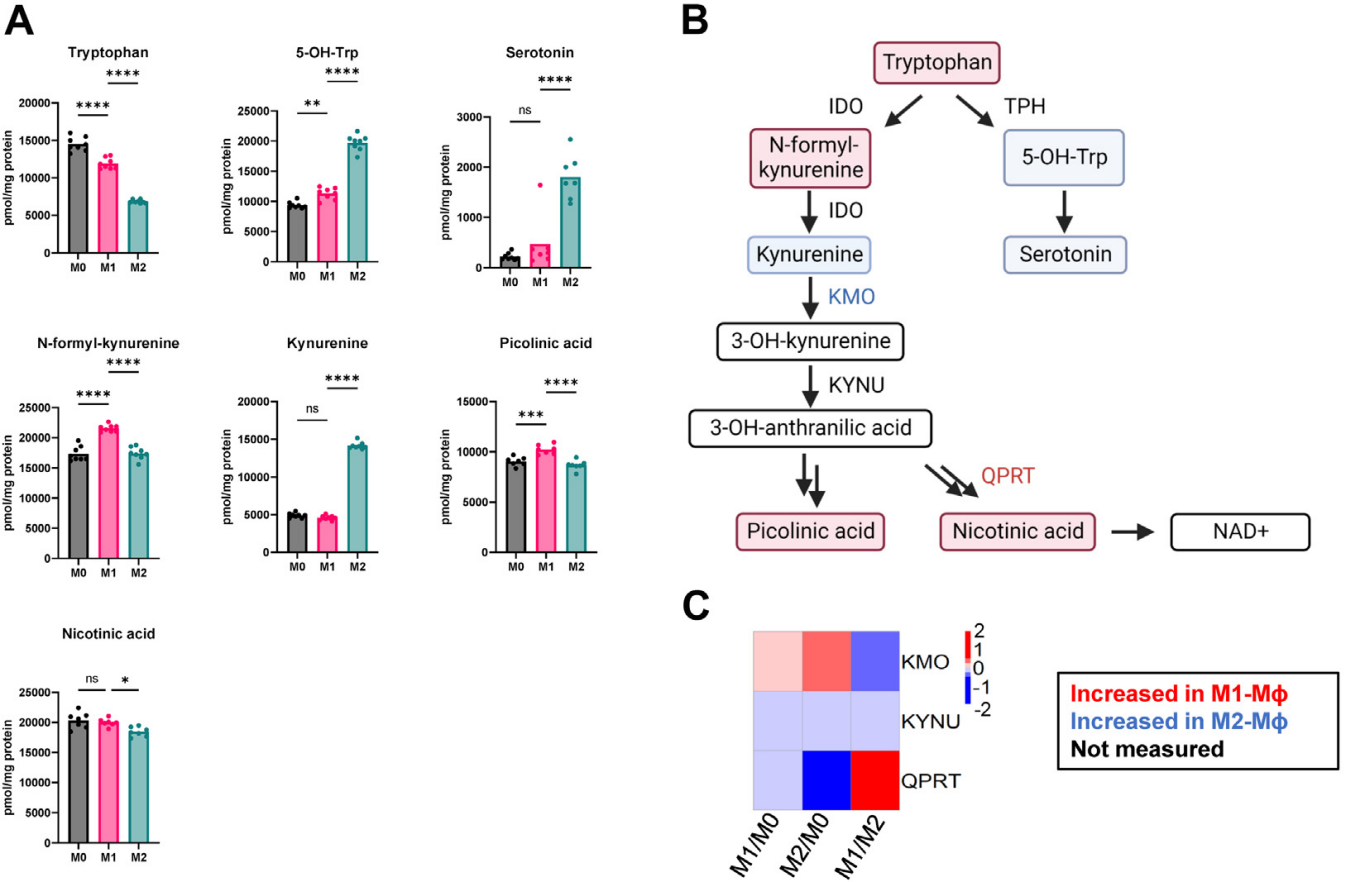
**Tryptophan metabolism in M1-Mɸ *versus* M2-Mɸs.***A*, concentrations of tryptophan pathway metabolites in M0-, M1-, and M2-MФs measured by LC-MS. Concentrations were calculated by normalizing the signals of targeting compounds against the signal of 10 μl of 0.1 mM ^13^C_2_, 2,2-^2^H_2_, ^15^N-Glycine spiked in 200 μl of acetone extracts*. B*, tryptophan pathway showing metabolites framed in *red* or *green* colors indicating an increase in either M1-MФs or M2. IDO: indoleamine 2,3-dioxygenase, TPH: tryptophan hydroxylase, KMO, kynurenine 3-monooxygenase; KYNU, kynureninase; QPRT, quinolinate phosphoribosyltransferase. *C*, heat map of the expressions of proteins identified in the tryptophan pathway.

Tryptophan can be metabolized by tryptophan hydroxylase to form 5-hydroxyl-tryptophan and later form serotonin. Alternatively, indolamine-2,3-dioxygenase 1 can metabolize tryptophan to form N-formyl-kynurenine and ultimately, kynurenine. Indolamine-2,3-dioxygenase 1 has antibacterial and immunosuppressive functions (59). Notably, its product, kynurenine, can mediate antimicrobial functions (60). We found that serotonin concentration was several fold lower in M0- and M1-MФs than in M2-MФ (Fig. 5*A*). This could be due to the inhibition of tryptophan hydroxylase by NO (Fig. 5*B*) (61). A higher level of serotonin in M2-MФ is consistent with earlier reports that serotonin has anti-inflammatory effects and suppresses the release of TNF-α and IL-1β in immune cells and IFN-γ–induced phagocytosis at high concentrations (62, 63, 64).

Kynurenine was dramatically increased in M2-MФs compared to M0- and M1-MФs (Fig. 5*A*). Kynurenine is known to act on the aryl hydrocarbon receptor, which can dampen the immune system response (65). Kynurenine can be further metabolized into downstream products, including NAD^+^, a critical energy metabolite for redox reactions, and it serves as the substrate for Sirtuin histone-modifying enzymes. We also detected other products of tryptophan metabolism, including picolinic acid, which was only moderately increased in M1-MФs (Fig. 5, *A* and *B*). We quantified three proteins in the tryptophan metabolic pathway, including kynurenine 3-monooxygenase (KMO), kynureninase (KYNU), and quinolinate phosphoribosyltransferase (QPRT) (Fig. 5*C*). KMO expression was decreased by 25% in M1-MФs compared to M2-MФs; KYNU expression was comparable, but QPRT expression increased 4-fold in M1-MФs compared to M2-MФs (Fig. 5*C*).

*De novo* NAD^+^ biosynthesis requires QPRT, which was decreased in M2-MФs. However, M2-MФs have higher levels of kynurenine and serotonin but lower levels of QPRT protein. This indicates the complex regulation of tryptophan metabolism at the proteomic and metabolomic level. High levels of kynurenine in M2-MФs might suggest increased NAD^+^ production as there may be increased utilization of the *de novo* synthesis pathway. However, M1-MФs may simply have a higher flux through the *de novo* NAD^+^ pathway and more readily produce NAD^+^ as an end product. M2-MФs, on the other hand, may accumulate these precursors, which are believed to dampen immune responses (65), at the cost of producing NAD^+^. Our initial study here cannot determine which possibility is correct, although the latter explanation would be consistent with the elevated histone acetylation in M2-MФs, as a lack of NAD^+^ would prevent Sirtuins from deacetylating histones. Further studies directly measuring NAD^+^ as well as using isotope tracing studies with tryptophan would be required to fully understand the utilization of this pathway.

### Central carbon metabolism and histone acetylation

**Figure 6:**
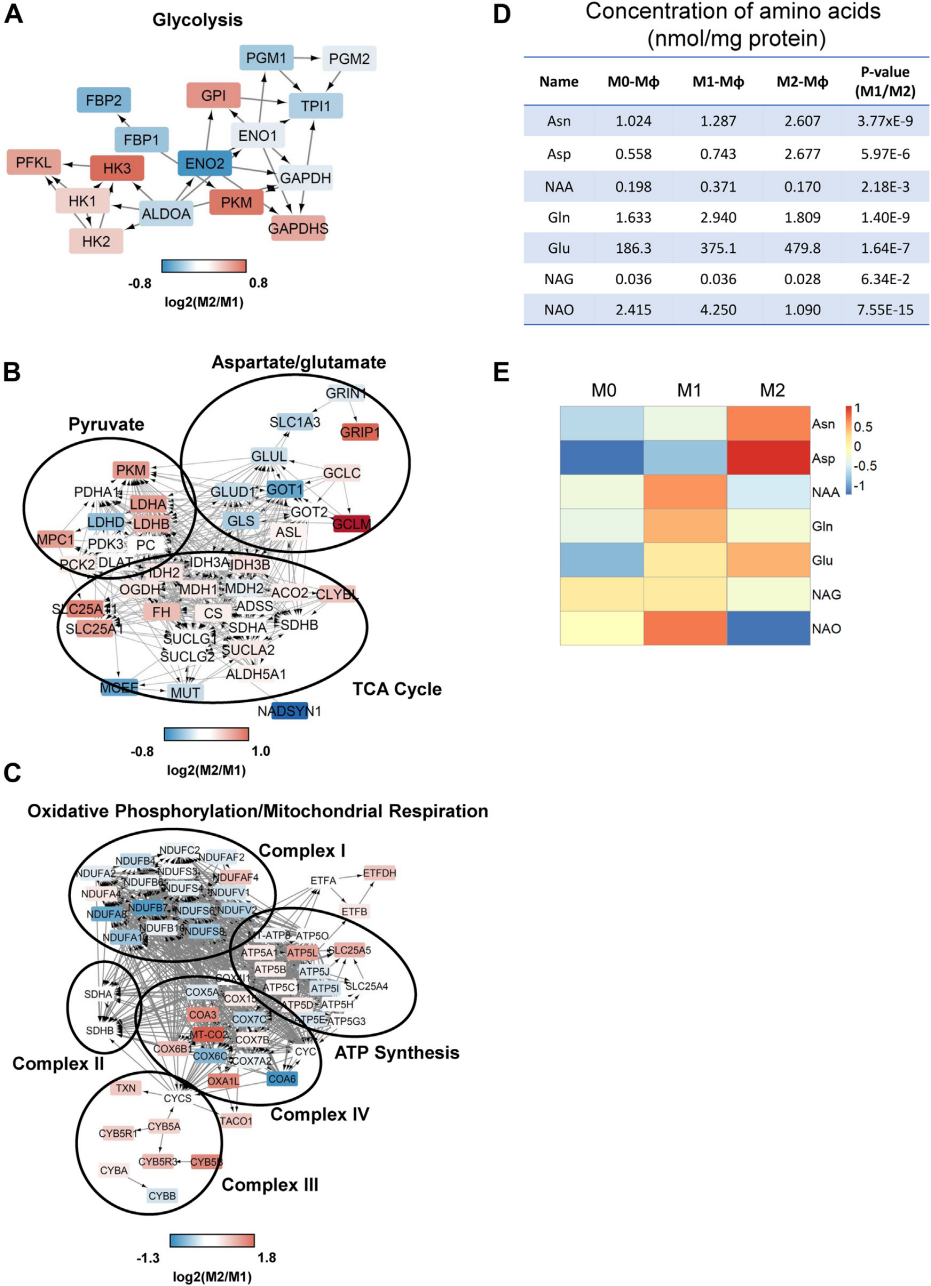
**Protein and metabolite alteration in glycolysis, TCA cycle, mitochondria respiration, and aspartate/glutamate to pyruvate shunting pathways in M1-Mɸ *versus* M2-Mɸs.***A*, STRING network of proteins detected by quantitative proteomics in glycolysis. *Red color*: proteins are upregulated in M2-MФ; *blue color*: proteins are upregulated in M1-MФ. LOG_2_ (fold changes) of protein expression (M2-MФ/M1-MФ) are represented by the color key on the bottom. *B*, STRING network of proteins detected by quantitative proteomics in mitochondria respiratory chain. *C*, STRING network of proteins detected by quantitative proteomics in TCA cycle, pyruvate, and aspartate/glutamate pathways. *D*, concentrations of asparagine, aspartic acid, glutamine, glutamic acid, acetylated aspartic acid, acetylated glutamic acid, and acetylated ornithine measured by LC/MS. *E*, heat map of (*D*). TCA, tricarboxylic acid.

Central carbon metabolism refers to the metabolic pathways, including glycolysis, the TCA cycle, and the electron respiratory chain. We successfully quantified nearly all catalytic enzymes in glycolysis using proteomics (Fig. 6*A*). The following glycolytic enzymes were elevated in M1-MФs: phosphoglucomutase 1 (PGM1), triosephosphate isomerase 1 (TPI1), fructose-bisphosphatase 1/2 (FBP1 and FBP2), fructose-bisphosphate aldolase A (ALDOA), and enolase 2 (ENO2). Alternatively, the following glycolytic enzymes were elevated in M2-MФs: hexokinase 1/2/3 (HK1, HK2, and HK3), liver type-phosphofructokinase (PFKL), glucose-6-phosphate isomerase (GPI), pyruvate kinase 2, glyceraldehyde-3-phosphate dehydrogenase spermatogenic. Levels of PGM2, ENO1, and glyceraldehyde-3-phosphate dehydrogenase were comparable between the two states. Figure 6*B* displays altered expression profiles of proteins in the TCA cycle and its coupled pyruvate and aspartate/glutamate pathways.

Except for citrate synthase (CS) and malate dehydrogenase 2 (MDH2), which were not differentially expressed, all TCA cycle enzymes detected were upregulated in M2-MФs. The major enzymes involved in pyruvate metabolism, pyruvate kinase M2 and lactate dehydrogenase A/B (LDHA and LDHB) were upregulated in M2-MФs, except lactate dehydrogenase D (LDHD), which was upregulated in M1-MФs. These results are consistent with prior reports of elevated TCA pathway in M2-MФs.

While glucose is the typical source of carbons for the TCA pathways, glutamate can also be used to fuel the TCA cycle. In M1-MФs, we found increased levels of glutamic-oxaloacetic transaminase 1 (GOT1), glutamate dehydrogenase 1 (GLUD1), glutamate-ammonia ligase (GLUL), glutaminase (GLS), and solute carrier family 1 member 3 (SLC1A3). These results suggest that M1-MФs may upregulate glutamine/glutamate incorporation into the TCA cycle rather than glucose.

Consistent with the elevated TCA enzymes in M2-MФs, we generally observed a corresponding increase in the enzymes involved in oxidative phosphorylation. Figure 6*C* displays altered expression of mitochondrial respiration chain proteins. Mitochondrial electron transport chain (ETC) complex I proteins were unchanged, except for NADH:Ubiquinone Oxidoreductase Complex Assembly Factor 4 (NDUFAF4), which was upregulated in M2-MФs. ETC complex II enzymes, succinate dehydrogenase A and B were comparable between M1-MФs and M2-MФs. Five members of ETC complex III were upregulated in M2-MФs, including cytochrome B (CYB)-A, 5A, 5B, 5R1, and 5R3. In contrast, CYBB was solely upregulated in M1-MФs. Elevated CYBB, also called NOX2, is consistent with reports that it is a primary component of the microbicidal phagocyte oxidase system and generates ROS in M1-MФs (66). The following cytochrome C oxidases (COXs) of ETC complex IV were upregulated in M2-MФs: COX2 (MT-CO2) fragment, COX5B, COX15, COA3 (COX assembly factor 3), OXA1L (COX assembly 1-like), and TACO1 (translational activator of COX1). On the other hand, COX5A, COX6C, COX7C, and COA5 (COX assembly factor 5) were upregulated in M1-MФs. ATP synthases are also differentially expressed: ATP5A/B/C/D/L were upregulated in M2-MФs, whereas ATP5E/G3/I/J were upregulated in M1-MФs.

All metabolites related to the TCA cycle and its coupled pyruvate and aspartate/glutamate pathways were measured using the O-benzylhydroxylamine (O-BHA) derivatization LC-MS/MS method. Pyruvate (Pyr) and lactate (Lac) concentrations were significantly higher in M1-MФs. We also found decreased citrate and α-ketoglutarate (α-KG) in M1-MФs. However, citrate/isocitrate isoforms were not fully resolved by our LC/MS method. These findings are consistent with previous reports that the TCA cycle is blocked before α-KG formation, by inhibition of either IDH or aconitase 2 (ACO2) *via* NO inhibition (19, 67). No differences in succinate or malate concentrations were found in M1-MФs and M2-MФs (Fig. S2). However, oxaloacetate (OAA) was elevated in M1-MФs (Fig. 2*A*), but OAA can be derived from aspartate or glutamate (68). Both aspartate and glutamate were significantly higher in M2-MФs compared to M1-MФs (Fig. 6, *D* and *E*). This may indicate that they accumulate in M2-MФs but are used in M1-MФs to form OAA for entry into the TCA cycle.

### Accumulation of acetylated amino acids in M1-MФs

Acetylated aspartic acid (N-acetyl-aspartic acid, NAA) and acetylated glutamate (N-acetyl-glutamate, NAG) were elevated in M1-MФs relative to M2-MФs (Fig. 6, *D* and *E*). The concentration of NAA was 10 times that of NAG, suggesting that NAA is the major pool of acetyl groups. The measured NAA concentration (∼0.4 nmol/mg protein in M1-MФs and ∼0.2 nmol/mg protein in M0-MФs and M2-MФs) was comparable to the concentrations measured in immortalized brown adipocytes (0.1 nmol/mg protein) (69, 70, 71, 72). Surprisingly, N-acetyl-ornithine (NAO) was also abundant (Fig. S3), and its concentration in M1-MΦs was about four times that of M2-MΦs and two times that of M0-MΦs (Fig. 6, *D* and *E*). NAO is the metabolic product of NAG (73). We propose that higher levels of NAA, NAG, and NAO in M1-MΦs may function as acetyl-CoA trap that could result in the observed decreases in histone acetylation (Fig. 2*B*).

### Tracking acetyl groups into histone acetylation

**Figure 7:**
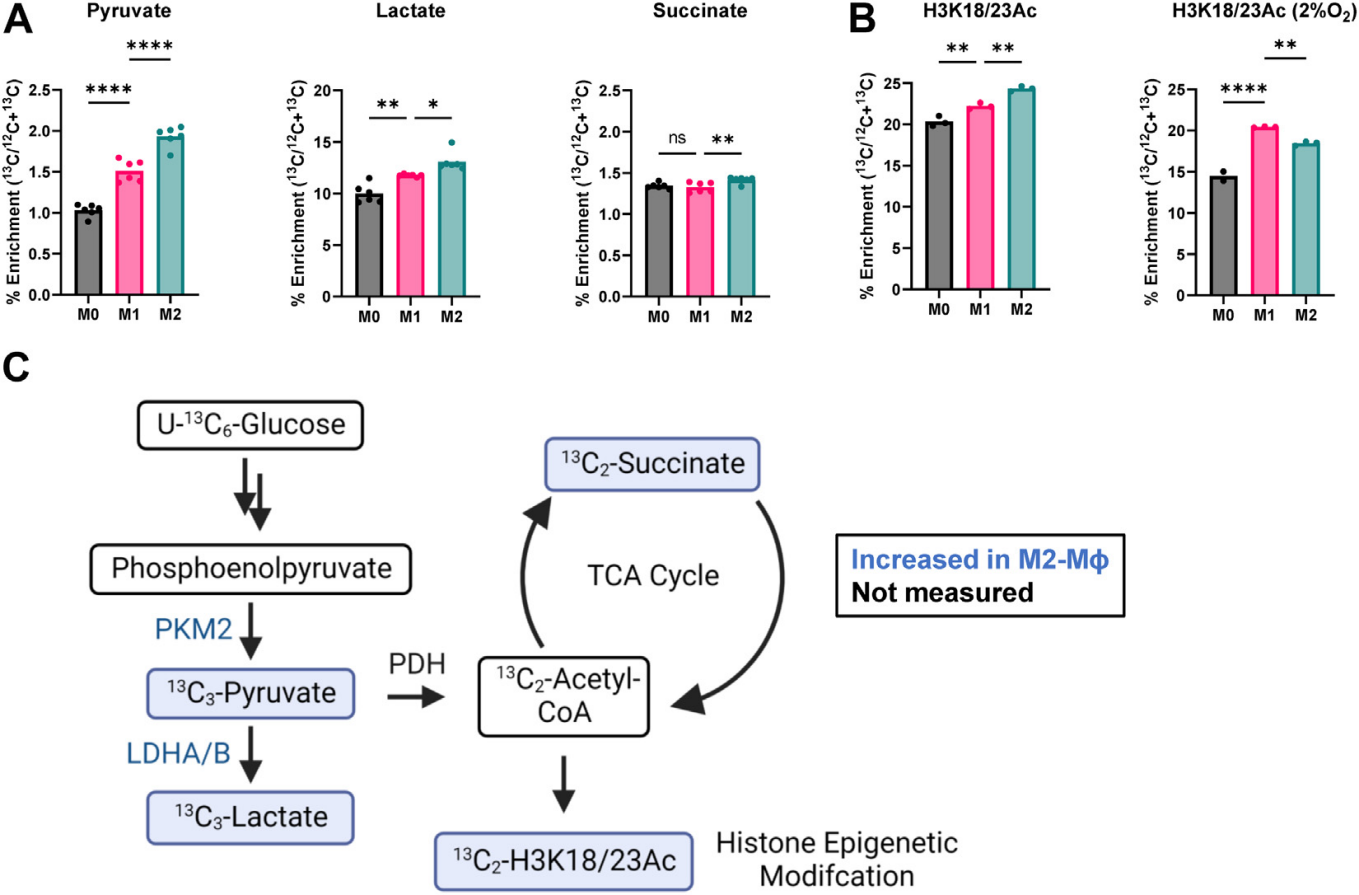
**Glucose tracing into key metabolites and histone acetylation.***A*, ^13^C3-enrichment of pyruvate and lactate and ^13^C_2_-enrichment of succinate from ^13^C_6_-glucose metabolism. *B*, percentage of ^13^C_2_-acetyl enrichment of histone H3K18/K23 after MФs incubated with ^13^C_6_-glucose for 48 h under normoxic and hypoxic conditions. *C*, diagram of ^13^C enrichment on glycolic and the TCA cycle metabolites and histone acetylation derived from ^13^C_6_-glucose in M1-Mɸ. TCA, tricarboxylic acid.

To better understand how glucose metabolism affects histone acetylation, we performed isotope tracing using uniformly labeled ^13^C_6_-glucose. Isotope enrichment of pyruvate and lactate was higher in M1-MФs than M0-MФs, confirming the upregulation of glycolysis by IFN-γ (Fig. 7*A*). However, levels of pyruvate and lactate were higher in M2-MФ than in M1-MФ. Unexpectedly the total isotope enrichment of pyruvate was only 1% to 2% while lactate was 10% to 15%. This would appear to indicate that pyruvate is being diverted to either lactate or acetyl-CoA. However, we see a similar 1% to 2% enrichment of succinate, suggesting acetyl-CoA is largely not being incorporated into the TCA cycle. Consistent with this, isotope-labeled glucose incorporation into H3K18/23 acetylation is enriched by 20% to 25%, which indicated that production of acetyl-CoA is not blocked but rather is diverted to histone acetylation rather than into the TCA cycle (Fig. 7*B*).

Decreased enrichment of succinate M^+2^ isotopologue (^13^C_2_) (Fig. 7*A*) confirms TCA cycle impairment at the formation of α-KG, which is also indicated by proteomics (Fig. 6*B*). ^13^C_2_-acetyl incorporation from glucose to histones was measured as illustrated by Fig. S4. Enrichment is higher in M1-MФs and further increased in M2-MФs, compared to M0-MФs, although the differences are relatively modest (Fig. 7*B*). These results are consistent with reduced global histone acetylation in M1-MФs compared to M2-MФs in nonisotope tracing experiments (Fig. 2*B*).

To examine the effects of hypoxia on histone modifications and isotope incorporation, MФs were cultured otherwise identically but under hypoxic conditions (2% O_2_). Levels of H3K18/23Ac isotope enrichment were decreased in M0-MФs and M2-MФs relative to normoxia. Consequently, the relative enrichment of this modification is higher in M1-MФs than either M0-MФs or M2-MФs under hypoxia. This observation is consistent with a previous report indicating that hypoxia suppresses metabolism through the TCA cycle by transactivating the gene encoding pyruvate dehydrogenase kinase 1 (*PDK1*). PDK1 then inactivates the pyruvate dehydrogenase complex (PDH), which results in decreased acetyl-CoA formation from glucose metabolism (74). Furthermore, one-carbon metabolism appeared to be inhibited in M1-MФs as there was reduced methyl transfer from serine to histones. Hypoxia further enhanced this effect (Fig. S5).

## Discussion

We have developed an innovative triomics method to understand the crosstalk between metabolism and histone modifications during IFN-γ and IL-4-mediated polarization of human MФs. From acetone extracts of cell lysate, we were able to quantify hundreds of metabolites containing primary amines or carboxylic acids using dansylation and O-BHA derivatization, respectively. The concentrations of the 20 amino acids, modified amino acids/dipeptides, such as methylated lysine and arginine, acetylated lysine and ornithine, arginine and glutamate metabolism, and tryptophan metabolism were successfully quantified by dansylation derivatization followed by LC-MS/MS analysis. Concentrations of TCA cycle intermediates were measured by O-BHA derivatization followed by LC-MS/MS analysis. We compared their relative concentration levels in M0-, M1-, and M2-MФs.

In M1-MФs, we saw increased levels of nitrotyrosine supporting their role in producing reactive nitrogen species to combat pathogens. Consistent with the ‘anti-inflammatory’ role of M2-MФs, tryptophan and arginine pathways revealed elevated levels of agmatine, serotonin, and kynurenine, which are all believed to play some anti-inflammatory role. Additionally, GSH detoxification was significantly upregulated and ROS formation is downregulated in M2-MФs (Fig. S6*A*). A corresponding decrease of 4-HNE (Fig. 2*A*), a marker of lipid peroxidation, as well as oxidized threonine (AAAS) and lysine (AKBA) was observed (Fig. S6, *B* and *C*). However, based on our results at the protein and metabolite level, previous reports that suggest a ‘glycolytic’ M1 subtype and an ‘Oxphos’ M2-subtype generalization may be just that, a generalization, and the dynamics of those pathways and their intersection with MФ polarization may be more complex than previously appreciated.

Interestingly, in all MФ subtypes studied herein, stable isotope tracing with glucose suggested that very little glucose made its way into the TCA cycle (Fig. 7, *A* and *C*). While the proteomic data suggested that M2-MФs may upregulate Oxphos, it appears that MФs overall do not utilize glucose molecules in the TCA cycle. It is possible that the enrichment of succinate is poor, not because the TCA cycle is dysfunctional, but rather that other sources of carbons, such as glutamate, are being preferentially used.

LC-MS/MS analysis demonstrated that histone acetylation was significantly lower in M1-MФs than M2-MФs (Fig. 2*B*). Our metabolomics data identified an accumulation of NAA, NAG, and the NAG metabolic product NAO in M1-MФs, which may explain the decreased levels of histone acetylation relative to M2-MФs. Although differential expression of histone acetyltransferases and deacetylases cannot be ruled out, lower acetyl-CoA production from glycolysis and blocked acetyl release from NAA, NAG, and NAO could also explain the epigenetic differences in polarized MФs.

Importantly, *de novo* synthesis of NAD^+^ through tryptophan metabolism was significantly upregulated in M1-MФs, as evidenced by elevated levels of nicotinic acid and significant overexpression of QPRT (Fig. 5). We predict that the production of NAD^+^ by oxidation of NADH *via* mitochondrial respiratory complex I is enhanced in M1-MФs because most complex I proteins were upregulated (Fig. 6*C*). Elevated NAD^+^ from tryptophan metabolism as well as NADH oxidation may, in turn, enhance the activity of sirtuin-type histone deacetylases, thereby reducing histone acetylation. Our metabolomic approach did not measure NAD^+^ levels and only provides a snapshot of tryptophan metabolism. Future studies should be done to investigate the mechanism by which we identified higher levels of kynurenine in M2-MФs but higher levels of downstream metabolites, closer toward NAD^+^ synthesis, in M1-MФs. Tryptophan metabolism may be a major contributor to the respective roles of M1-MФs and M2-MФs in immune activation.

Because acetyl-CoA can also be produced from fatty acid β-oxidation, we sought to determine whether fatty acid β-oxidation occurs differentially in M0-, M1-, and M2-MФs (Fig. S6). Our data suggest that there are no significant differences in fatty acid β-oxidation among the three types of MФs. Concentrations of middle to long chain fatty acids, including hexanoic acid, palmitic acid, and palmitoleic acid, were significantly lower in M1-MФs. If these were degraded by β-oxidation to short chain fatty acids, we should have detected considerably higher concentrations of acetic and butyric acids. However, this was not found (Fig. S6, *B* and *C*). Middle to long chain fatty acids are likely consumed to synthesize very long chain fatty acids due to upregulation of ELOVL1 (Fig. S6*A*).

Methylated arginine derived from protein and/or histone degradation and their specific methyltransferases, may regulate MФ polarization. Dimethylated arginine-glycine (RG/GR) peptides were highly abundant and present in significantly higher quantities in M1-MФs relative to M2-MФs. Although further confirmation is needed, the data suggest RG/GR domain–containing proteins, such as H4-R3 and RelA (R30), are hypermethylated. Perhaps most importantly, N-mono-methyl-L-arginine and asymmetric dimethylated arginine are also known to modulate NO production by competing for the arginine-binding site of NOS. This creates a potential intersection of arginine metabolism, protein post-translational methylation, which is SAM/one-carbon metabolism dependent, protein degradation, and the modulation of NO production.

## Experimental procedures

### M1-MФs and M2-MФ cell culture, differentiation, isotope tracing, and hypoxia

Human peripheral blood–derived buffy coat of HIV negative and MTB negative healthy donors (male and females, deidentified) were purchased from the Gulf Coast Regional Blood Center/Bank in Houston (1400 La Concha Ln, Houston, TX 77054), which is exempt from IRB regulations of Houston Methodist Research Institute. Blood from about 10 donors was used in this study. All blood samples were processed per approved IRB (HSCA00000993) protocols of the Houston Methodist Research Institute. Procedures and phenotype/purity determination of peripheral blood mononuclear cell (PBMC)–derived and PBMC-differentiated macrophages have been previously described (29). Briefly, CD14^+^ magnetic bead (Miltenyi Inc) purified monocytes from PBMCs were grown in Iscove's medium (IMDM) with 10% fetal bovine serum and 10 ng/ml granulocyte-macrophage colony stimulating factor (GM-CSF) at 4 × 10^6^ per well in 6-well tissue plates for 6 days and then rested for 24 h in GM-CSF–free medium. Cells were differentiated using IFN-γ (10 ng/ml) or IL-4 (10 ng/ml) in GM-CSF–free medium for 72 h to obtain M1-MΦs and M2-MΦs, respectively. M0-MФs were not treated with cytokines. For isotope tracing experiments, M0-, M1-, and M2-MΦs were washed once with PBS and then incubated in RPMI-1460 medium with stable isotope-labeled 2 g/L D-glucose (U-^13^C_6_, 99%, Cambridge, catalog no.: #CLM-1396-1) and 200 μM L-serine (2,3,3-D_3_,98%, Cambridge, catalog no.: #DLM-582-0.5) for an additional 2 and 3 days, respectively. IFN-γ (10 ng/ml) or IL-4 (10 ng/ml) were added again for M1-MΦs and M2-MΦs. Separated sets of M0-, M1-, and M2-MΦs were cultured and treated simultaneously under hypoxic conditions (2% O_2_).

### Triomics approach

Triomics refers to 'metabolomics, proteomics, and OMICs of histone modifications'. We integrated these three OMICs seamlessly in a one sample preparation followed by an analysis of metabolites, histone modifications, and proteins on the QExactive mass spectrometer (Fig. 1). Briefly, cell pellets of M0-, M1-, and M2-MФs were lysed using radioimmunoprecipitation assay buffer (ThermoFisher Scientific, catalog no.: #89901) supplemented with 1% Nonidet P40, PMSF (0.2 mM), and Roche complete protease inhibitor cocktail (ThermoFisher Scientific, catalog no.: #50-100-3301, one tablet per 10 ml lysis buffer). The protein concentrations were measured by bicinchoninic acid assay. An aliquot (∼100 μl) of lysate containing 100 μg proteins was immersed in 500 μl of precooled (−20 °C) acetone overnight. After centrifugation, metabolites in the supernatant were submitted for metabolomics analysis, and the proteins in the precipitates were used for the analysis of histone modifications and differential proteins expression.

### Analysis of primary amines including amino acids by dansylation derivatization followed by nano-LC-MS/MS analysis

Primary amine compounds, including amino acids, were derivatized by dansylation before analysis by nano-LC/MS based on a method (75, 76) and adapted to our nanoLC-QExactive systems. Briefly, 100 μl acetone extraction was mixed with 10 μl internal standards containing 0.1 mM ^13^C_2_, 2,2-^2^H_2_, ^15^N-Glycine (Cambridge isotope Lab, catalog no.: #CDNLM-6799-0.25), 0.1 mM ^13^C_3_, ^2^H_3_, ^15^N-Serine (Cambridge isotope Lab, catalog no.: #CDNLM-6813-0.25) and 0.1 mM Methyl-D_3_-Methionine (Cambridge isotope Lab, catalog no.: #DLM-431-1), vacuum-dried, and then 30 μl of 0.5 M Na_2_CO_3_/NaHCO_3_ and 100 μl of dansyl chloride (4 mg dissolved in 1 ml acetone) was added and incubated at 55 °C for 1 h. After drying under vacuum, dansylated metabolites were dissolved in 50 μl 88% formic acid and 50 μl 1% formic acid. A 20 μl aliquot was desalted by C_18_ desalting columns. After removal of acetonitrile, which was used to elute the dansylated compounds, eluates were dissolved in 50 μl 40% methanol in 1% formic acid. This solution was further diluted 10× with 1% formic acid. Two microliters of this solution was injected each time through the autosampler and analyzed by nanoLC-MS/MS on the QExactive benchtop orbitrap mass spectrometer. The HPLC column was made in-house by packing Magic C_18-_AQ (100 Ǻ, 3 μm, Bruker, catalog no.: #PM3/61100/00) package material into 20 cm (length) × 360 μm (outer diameter) × 75 μm (internal diameter) capillary with PicoTip emitter (New Objective, catalog no.: #PF360-75-15-N-5) under high pressure. Using this method, amino acids, serine, glycine, and methionine were accurately measured using their stable isotope-labeled compounds as internal standards, and concentrations of other compounds were estimated by MS signal normalization against that of either serine in the low retention time zone or glycine in the high retention time zone.

### Analysis of carbonyl-containing compounds including TCA intermediates by O-BHA derivatization followed by nano-LC-MS/MS analysis

A previously reported method was adapted (77). Acetone extracts (200 μl), with added 10 μl mixture of internal standards containing 0.1 mM each of sodium D_3_-pyruvate (Cambridge isotope Lab, catalog no.: #DLM-6068-0), sodium ^13^C_3_-L-lactate (Cambridge isotope Lab, catalog no.: #CLM-1579-N-0.1MG), 1,5,6-^13^C_3_-citric acid (Cambridge isotope Lab, catalog no.: #CLM-9876-0.1MG), 1,2,3,4-^13^C_4_-α-ketoglutaric acid disodium salt (Cambridge isotope Lab, catalog no.: #CLM-4442-0.1MG), ^13^C_4_-succinic acid (Cambridge isotope Lab, catalog no.: #CLM-1571-0.1MG), ^13^C_4_-fumaric acid (Cambridge isotope Lab, catalog no.: #CLM-1529-0.1MG), and ^13^C_4_-L-malic acid (Cambridge isotope Lab, catalog no.: #CLM-8065-0.1MG) were completely dried under vacuum. In the dried vials, 50 μl of 1-ethyl-3-(3-dimethylaminopropyl) carbodiimide, 100 μl of O-BHA in pyridine buffer (preparation [200 ml]:15 ml of pyridine (99.8%, Sigma) + 10 ml HCl (12 N) and 175 ml H_2_O) were added sequentially and then incubated at 30 °C for 1 h. After derivatization, metabolites were extracted by 2 × 300 μl ethyl ester. After vacuum drying, 200 μl of 40% methanol was added to dissolve the metabolites. The solution was further diluted 10× with 1% formic acid and submitted for nano-LC-MS/MS analysis on the QExactive. Using this method, carboxylic acids were analyzed. The concentrations of TCA intermediates were accurately measured using their stable isotope-labeled compounds as internal standards, whereas others were estimated by mass spectrometric signal normalization against malate.

### Analysis of histone modifications and the transferring of acetyl groups from metabolites to histones

An aliquot of cell lysate containing 20 μg of total proteins was mixed with 1 μg of SILAC-produced heavy arginine (^13^C_6_^15^N_4_, aka, ^10^R) histones (purity > 99%) before being loaded onto SDS-PAGE gel for electrophoresis separation. Gel bands covering molecular weights from 9 to 20 KDa containing histones were cut for in-gel digestion with trypsin. Quantification of histone modifications was carried out by parallel reaction monitoring on the QExactive instrument using a previously reported inclusion list (78, 79). The ratios of MS peak areas of modified peptides over histone peptides without modifications were calculated as the modification percentages used for comparison among M0-, M1-, and M2-MФ samples. For monitoring methyl transfer from 3,3,2-^2^H-serine or acetyl from ^13^C_6_-glucose to histones, except that no ^10^R-histones were added as internal standards. In this case, relative ratios of the ion intensities of isotope-labeled ^13^C_2_-acetyl-lysine peaks over those of unlabeled peaks were calculated as the isotope incorporation (or transfer) rates.

### Analysis of protein expression

Cell lysates of each sample containing 50 μg of proteins were reduced by adding 200 μM tris(2-carboxyethyl) phosphine and incubating at 55 °C for 1 h, followed by carbamidomethylation of cysteines by adding 375 mM iodoacetamide, and incubating the mixture in the dark for 30 min. Proteins were precipitated by adding six volumes of precooled acetone and freezing at −20 °C overnight and then pelleted at 13,000 rpm for 10 min at 4 °C. The protein pellets were then dissolved in 100 mM triethylammonium bicarbonate buffer followed by digestion with trypsin/Lyc-C (ThermoFisher Scientific, catalog no.: #A40007) at a protein/enzyme ratio of 25:1. A tandem mass tag (TMT) labeling kit (TMTsixplex (TMT6)) (ThermoFisher Scientific, catalog no.: #90064) was used to label the peptides according to manufacturer instructions. All of the indicated chemicals and solutions were included in this kit unless otherwise specified. M0-, M1-, and M2-MФ samples were labeled with TMT6-126 and TMT6-127, TMT6-128 and TMT6-129, and TMT-130 and TMT-131, respectively. After labeling and quenching, the six samples were desalted by Pierce graphite spin columns (ThermoFisher Scientific, catalog no.: #88302) and then mixed. The peptide mixture was fractionated into eight fractions with the Pierce high pH reversed-phase peptide fractionation kit (ThermoFisher Scientific, catalog no.: #84868) and dried by vacuum centrifugation. Each fraction was resuspended in 25 μl 1% formic acid and analyzed by nano-LC-MS/MS on the QExactive. The same column used for metabolite analysis was used for peptide separation. Reversed-phase liquid chromatography was run for 230 min (solvent A, 0.1% formic acid in water; solvent B, 0.1% formic acid in acetonitrile). A gradient of 5% to 45% of solvent B was used for peptide separation. The QExactive was set to acquire data at a resolution of 35,000 in full scan mode and 17,500 in MS/MS mode. The top 15 most intense ions in each MS survey scan were automatically selected for MS/MS. The liquid chromatography and MS running conditions (settings) were very similar for metabolites and peptides.

### Data analysis and statistical analysis

Each sample was a pool of cell pellets from three wells of a 6-well plate (3 × 4 × 10^6^ cells). At least eight injections of LC-MS/MS were performed for every sample analyzed. Metabolomics data acquired from QExactive instrument were processed by Xcalibur. Metabolite peak areas were integrated after manually inputting the calculated metabolites of interest. Less than 5 ppm between calculated mass and measured mass was considered an authentic identification; however, in most cases, identification was further verified by fragmentation patterns consistent with the molecule's chemical structure. A two-tailed Student's *t* test was used to test for the significance of metabolic changes between M1-MФ and M2-MФ for the volcano plot visualization. Otherwise for all bar graphs shown a one-way ANOVA was used with Dunnett’s test for multiple comparisons to the M1-MФs with GraphPad PRISM (GraphPad Software Inc). *p*-values < 0.03 are denoted ∗, <0.002 ∗∗, < 0.0002 ∗∗∗, < 0.0001 ∗∗∗∗.

Histone modifications were evaluated by the percentage (or ratio) of the peak area of a modified peptide over a peptide free of modifications, which were normalized to the percentage (or proportion) of the same pair of peptides with stable isotope labeling at arginine (79). Two-tailed Student's *t* test of the Excel-imbedded T.TEST function was used to test for the significance of modification changes between M1- and M2-Mɸ.

Proteins were identified with the Proteome Discoverer (PD) 2.2 platform (version 2.2.0.388, Thermo Fisher Scientific) using the SEQUEST HT search engine that employs the nr_human_062321validated.fasta database with 420,779 protein sequence entries (downloaded on June 23, 2021). SEQUEST HT parameters were specified as trypsin enzyme, three missed cleavages allowed, minimum peptide length of four, precursor mass tolerance of 10 ppm, and a fragment mass tolerance of 0.02 Da. Oxidation of methionine, acetylation at N-terminal, TMT at lysine, and deamination of asparagine were set as variable modifications. Carbamidomethylation of cysteine and TMT at peptide N termini was set as a fixed modification. Peptides were filtered for a maximum false discovery rate of 1% (strict). Protein quantification was also through PD 2.2 using the reporter ion ratios of TMT for each set. Reporter ions were quantified from MS2 scans using an integration tolerance of 0.04 Da with the most confident centroid setting. At least one unique peptide with a posterior error probability of <0.05 was accepted for quantification and proteins were grouped. Differential expression between M1- and M2-MФs was done using significance analysis of microarrays with permutation-based multiple test correction in the Perseus software (maxquant.net/perseus/) (1.6.12.0) (80). The false detection rate was set to 0.01 (1%) and s0 set to 0.32 (∼1.25× fold change cutoff).

Protein interaction networks were built upon the list of proteins in gene names identified by proteomics whose expressions had significant changes using the STRING web-based software. Output coordinates were revisualized by Cytoscape with nodes colored from blue (negative Log_2_ fold change) to red (positive Log_2_ fold change) based on protein quantification data. Pathways corresponding to a group of proteins selected from proteomics data meeting defined criteria were identified by g:Profiler and visualized by Cytoscape with EnrichmentMap APP (50).

## Data availability

Data supporting the original contributions presented in the study are included in the article and supplementary materials. Proteomics raw data and searching results were deposited in ProteomeXchange with the identifier PXD030701.

## Supporting information

This article contains supporting information.

## Conflict of interest

The authors declare that they have no conflicts of interest with the contents of this article.
