# Supplementary figures and images for: Multi-OMICs analysis reveals metabolic and epigenetic changes associated with macrophage polarization
Source: J Biol Chem. 2022 Aug 27;298(10):102418. doi: 10.1016/j.jbc.2022.102418 (PMC9525912; doi:10.1016/j.jbc.2022.102418)

## Slide 1
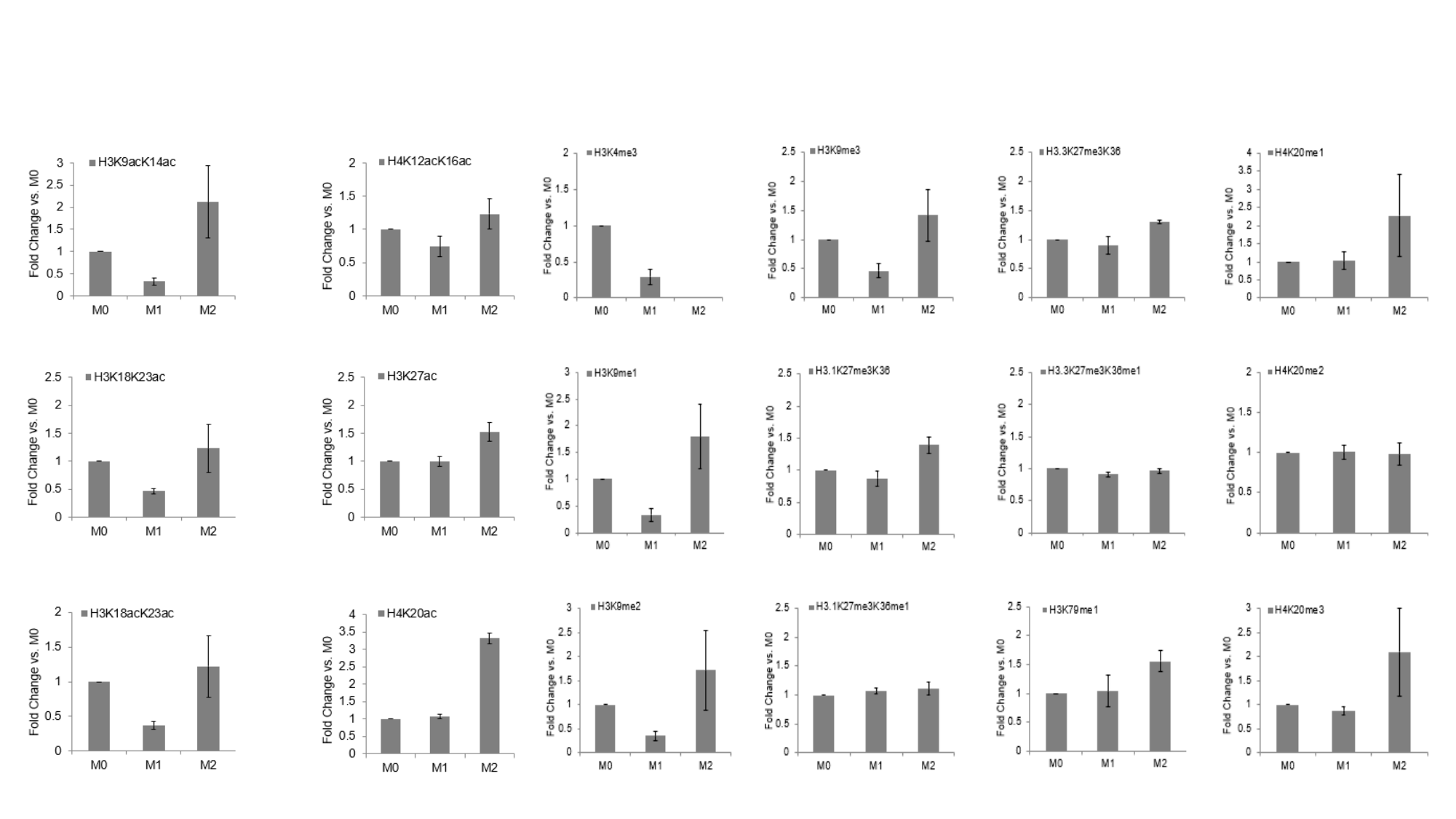

Supplement: Figure S1 [file mmc3.pptx]
