# Supplementary material for: Multi-OMICs analysis reveals metabolic and epigenetic changes associated with macrophage polarization
Source: J Biol Chem. 2022 Aug 27;298(10):102418. doi: 10.1016/j.jbc.2022.102418 (PMC9525912; doi:10.1016/j.jbc.2022.102418)
Supplement: Figure S2 [file mmc4.pptx]

## Slide 1
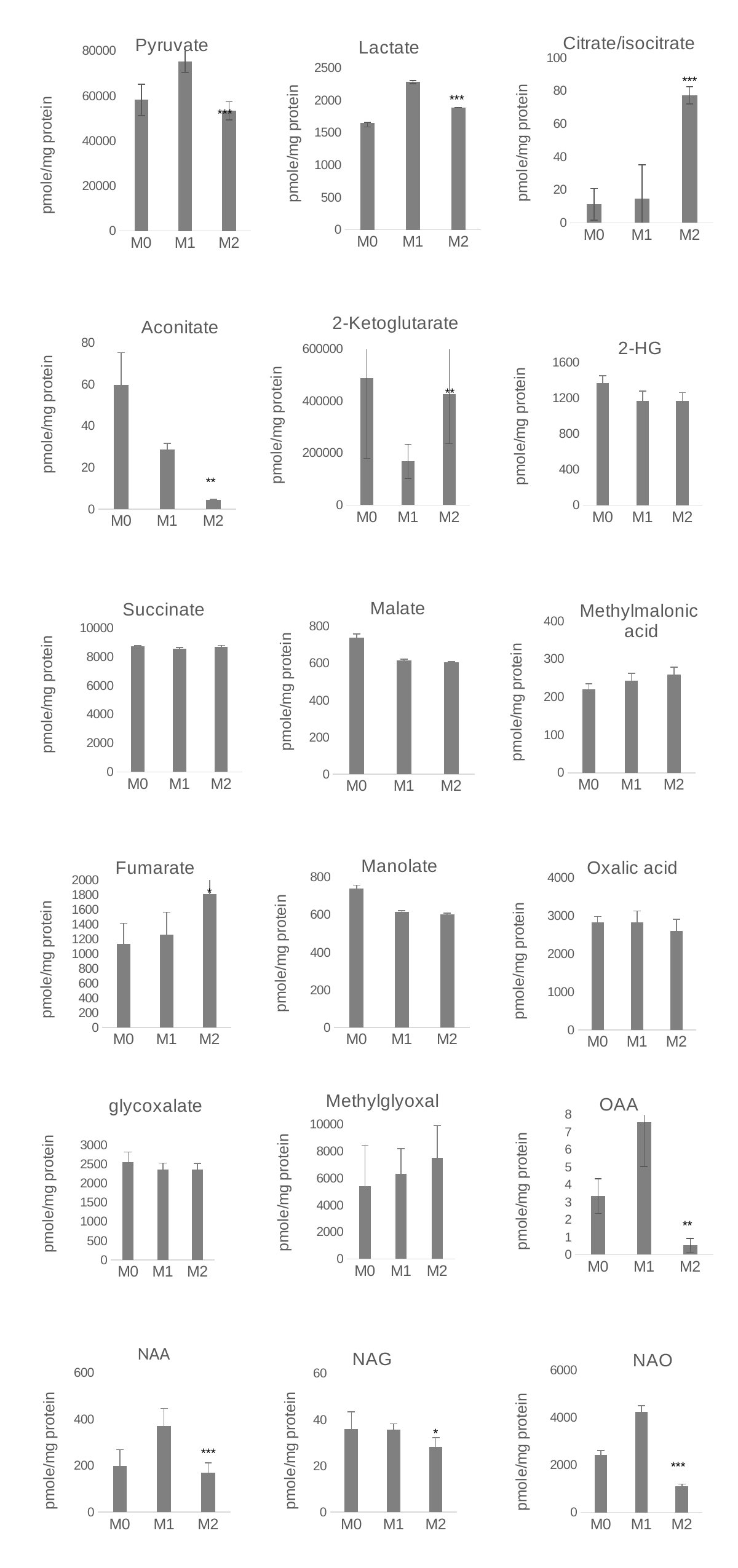

### Chart: Citrate/isocitrate
| Category | Citrate |
|---|---|
| M0 | 11.170643537745445 |
| M1 | 14.611624100743441 |
| M2 | 77.26399268752417 |
### Chart:
| Category | Pyruvate |
|---|---|
| M0 | 58220.02678289459 |
| M1 | 75259.96666423112 |
| M2 | 53364.954039095595 |
### Chart:
| Category | Lactate |
|---|---|
| M0 | 1650.8230253978847 |
| M1 | 2283.615030844372 |
| M2 | 1882.3560973929882 |***
***
***
### Chart:
| Category | 2-Ketoglutarate |
|---|---|
| M0 | 488309.63839859655 |
| M1 | 168467.75101649246 |
| M2 | 427401.51780843997 |
### Chart:
| Category | Aconitate |
|---|---|
| M0 | 59.79533354332427 |
| M1 | 28.72678631342293 |
| M2 | 4.544535202700546 |
### Chart: 2-HG
| Category | 2-Hydroxyglutarate |
|---|---|
| M0 | 1366.6233918884122 |
| M1 | 1167.2424116833852 |
| M2 | 1168.2933535043219 |**
**
### Chart: Succinate
| Category | Succinate |
|---|---|
| M0 | 8733.426800638312 |
| M1 | 8568.462038328222 |
| M2 | 8704.681004005406 |
### Chart: Methylmalonic
acid
| Category | Methylmalonic acid |
|---|---|
| M0 | 220.3083819381186 |
| M1 | 242.78752276689187 |
| M2 | 259.33598046392945 |
### Chart:
| Category | Malate |
|---|---|
| M0 | 739.6043992270394 |
| M1 | 617.5240544770093 |
| M2 | 604.4960801812648 |
### Chart:
| Category | Manolate |
|---|---|
| M0 | 739.6043992270394 |
| M1 | 617.5240544770093 |
| M2 | 604.4960801812648 |
### Chart:
| Category | Fumarate |
|---|---|
| M0 | 1138.2238908931006 |
| M1 | 1263.2434569740471 |
| M2 | 1809.4235254293058 |
### Chart:
| Category | Oxalic acid |
|---|---|
| M0 | 2828.9822269297606 |
| M1 | 2827.3475875624467 |
| M2 | 2606.231538921618 |*
### Chart:
| Category | OAA |
|---|---|
| M0 | 3.347298914669423 |
| M1 | 7.559045879475222 |
| M2 | 0.5289486456124313 |
### Chart: Methylglyoxal
| Category | Methylglyoxal (MG) |
|---|---|
| M0 | 5400.45471041482 |
| M1 | 6331.374453964331 |
| M2 | 7504.120556822142 |
### Chart:
| Category | glycoxalate |
|---|---|
| M0 | 2562.968115022761 |
| M1 | 2365.773947469914 |
| M2 | 2363.224880657589 |**
### Chart:
| Category | NAA |
|---|---|
| M0 | 197.78379832761416 |
| M1 | 370.9958659502509 |
| M2 | 170.2074039488728 |
### Chart:
| Category | NAG |
|---|---|
| M0 | 35.94667083818948 |
| M1 | 35.77507215709209 |
| M2 | 28.20056257035509 |
### Chart:
| Category | NAO |
|---|---|
| M0 | 2415.6810422765107 |
| M1 | 4250.01677387924 |
| M2 | 1089.8093569311707 |*
***
***
