# Supplementary material for: Multi-OMICs analysis reveals metabolic and epigenetic changes associated with macrophage polarization
Source: J Biol Chem. 2022 Aug 27;298(10):102418. doi: 10.1016/j.jbc.2022.102418 (PMC9525912; doi:10.1016/j.jbc.2022.102418)
Supplement: Figure S3 [file mmc5.pptx]

## Slide 1
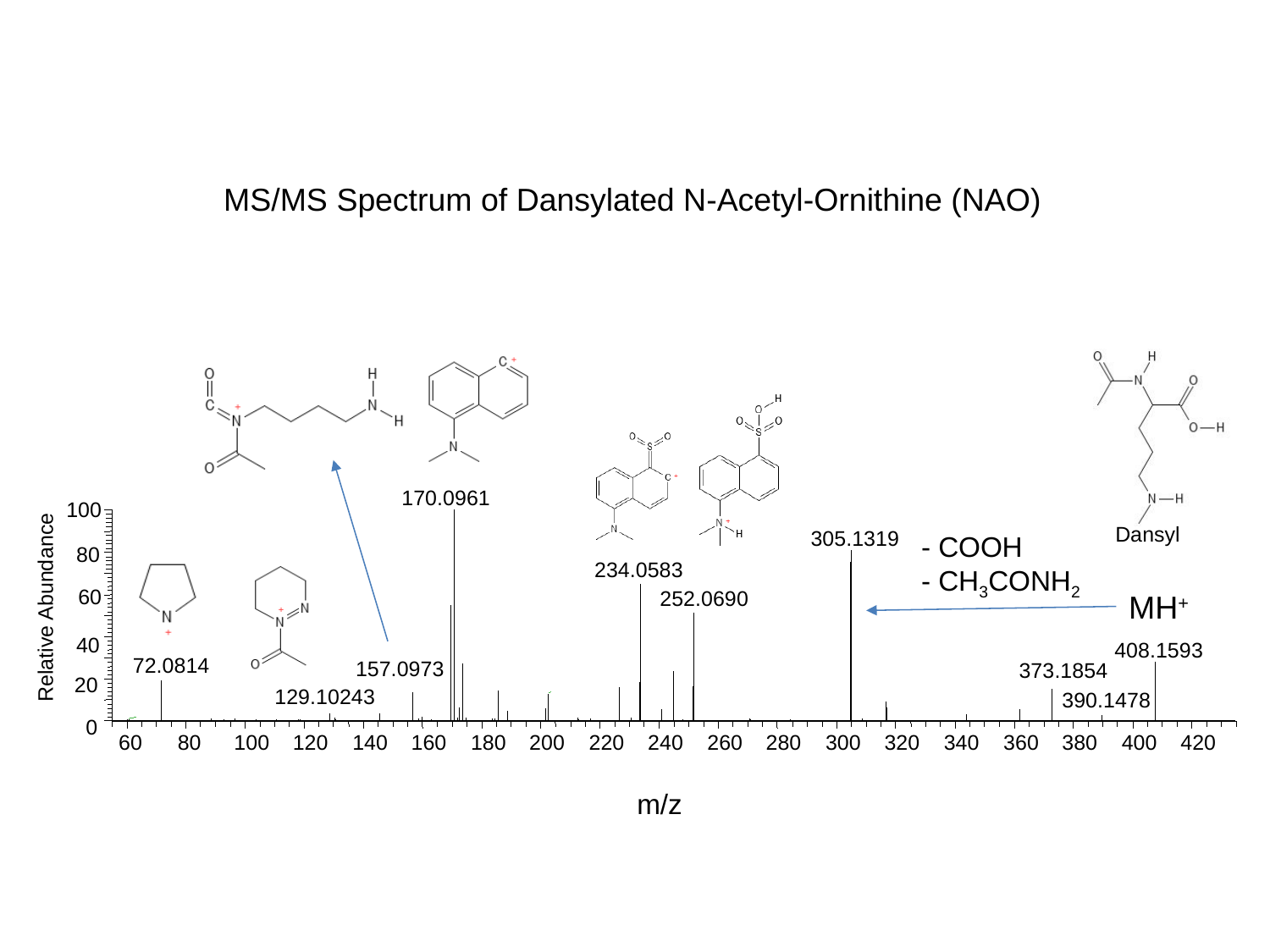

MS/MS Spectrum of Dansylated N-Acetyl-Ornithine (NAO)
60
80
100
120
140
160
180
200
220
240
260
280
300
320
340
360
380
400
420
m/z
170.0961
100
Dansyl
- COOH
- CH3CONH2
305.1319
80
234.0583
MH+
60
252.0690
Relative Abundance
40
408.1593
72.0814
157.0973
373.1854
20
129.10243
390.1478
0
