# Supplementary material for: Multi-OMICs analysis reveals metabolic and epigenetic changes associated with macrophage polarization
Source: J Biol Chem. 2022 Aug 27;298(10):102418. doi: 10.1016/j.jbc.2022.102418 (PMC9525912; doi:10.1016/j.jbc.2022.102418)
Supplement: Figure S4 [file mmc6.docx]

**SUPPLEMENTAL FIGURES**

**Figure S1**. Histone acetylation and methylation in M0-, M1-, and M2-MФs quantified by parallel-reaction-monitoring (PRM).

**Figure S2.** Concentrations of TCA cycle metabolites and N-acetyl-aspartate, -glutamate, and -ornithine measured by LC-MS/MS.

**Figure S3.** MS/MS spectrum of N-alpha-acetyl-ornithine dansylated derivative.

**Figure S4**: Measurement of acetyl transfer from metabolites to histones.

Rate of transferring of ^13^C_6_-glucose to histones: intensity of peak at *m/z* 128.0981 (the immonium ion of ^13^C_2_-acetylated lysine) over intensity of peak at *m/z* 126.0914 (the immonium ion of acetylated lysine). Shown is a selective MS/MS spectrum of K9me1K14ac.

**Figure S5. Altered one-carbon metabolism and histone methylation in M1- and M2-Mɸs.**

1. A network built up from one carbon metabolism (folate cycle and methionine cycle) proteins quantified by proteomics. Red color: Upregulated in M2-MФ; Blue color: upregulated in M1-MФ. Color key is the same for B.
2. Heat map of the relative expression (M2/M1) of the networked proteins.
3. The concentration ratio of methionine (Met) over homocysteine (Hcy) in M0-, M1-, and M2-MФs.
4. The concentration ratio of S-adenosyl-L-methionine (SAM) over S-adenosyl-homocysteine (SAH) in M0-, M1-, and M2-MФs.
5. Isotope tracing methyl transfer from 3,3,2-^2^H-serine to methionine (Met) and histone K9 mono- (H3K9me1) and tri-methylation (H3K9me3).
6. A scheme showing the isotope ^2^H transferring from serine to histones through the one-carbon pathway that is inhibited by NO.

**Figure S6**. Fatty acid metabolism and oxidation.

1. Networks built upon proteins quantified by proteomics in metabolic pathways of fatty acid oxidation and elongation, as well as production and detoxification of oxidative species. Red color: upregulated in M2-MФs. Blue color: upregulated in M1-MФs.
2. Concentrations (nmol/mg protein) of fatty acids and oxidized products in M0-, M1-, and M2-MФs. 4-HNE: 4-hydroxynonenal; AAAS: aminoadipic acid semialdehyde; AKBA: amino-ketobutyric acid.
3. Heat map showing the changes of the concentrations of fatty acids and oxidized products in B.
4. Networks built upon proteins quantified by proteomics in metabolic pathways of fatty acid oxidation and elongation, as well as production and detoxification of oxidative species. Red color: upregulated in M1-MФs. Blue color: upregulated in M0-MФs.
