# Supplementary material for: Multi-OMICs analysis reveals metabolic and epigenetic changes associated with macrophage polarization
Source: J Biol Chem. 2022 Aug 27;298(10):102418. doi: 10.1016/j.jbc.2022.102418 (PMC9525912; doi:10.1016/j.jbc.2022.102418)
Supplement: Figure S5 [file mmc7.pptx]

## Slide 1
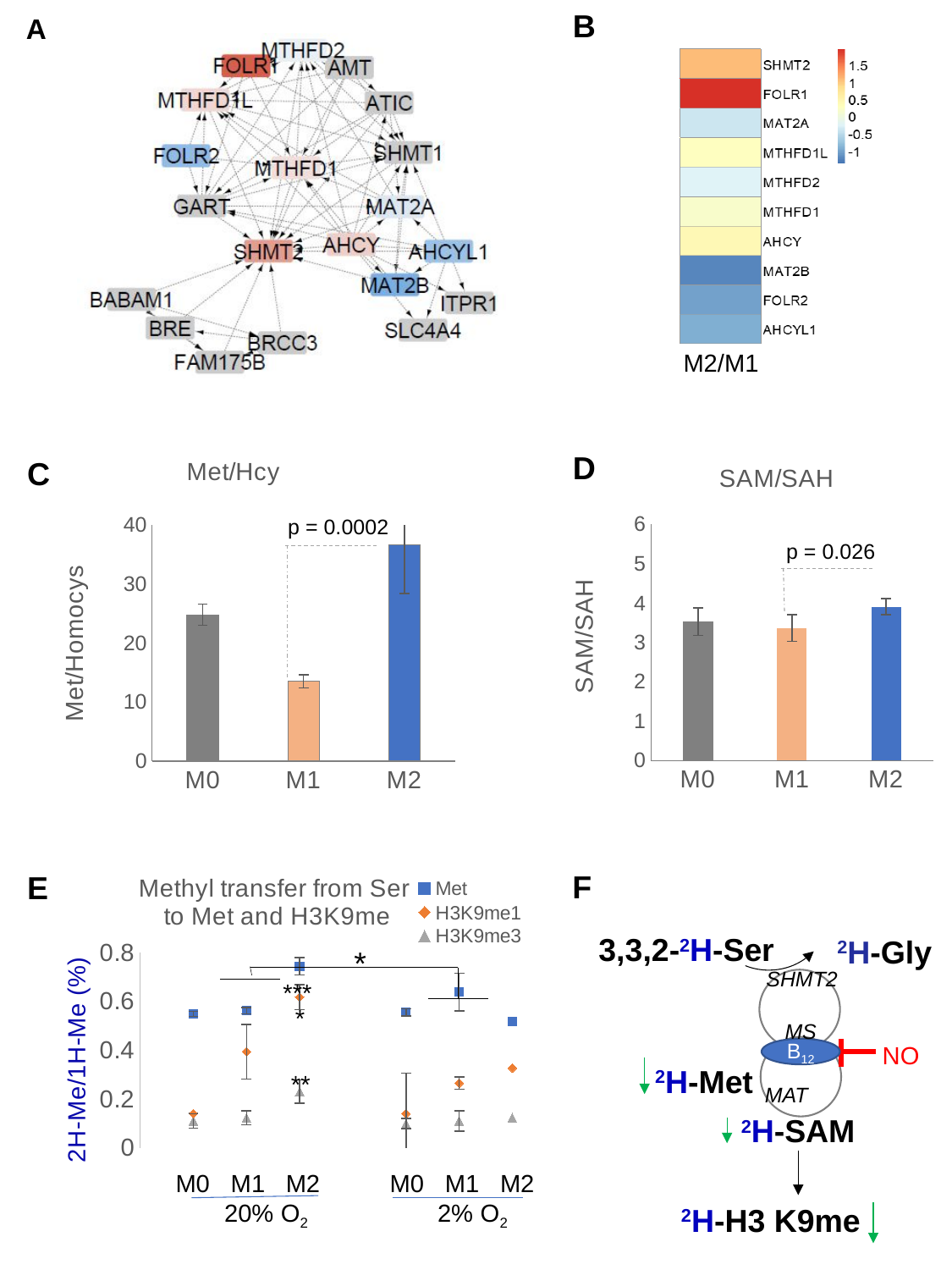

B
A
M2/M1
D
C
### Chart:
| Category | SAM/SAH |
|---|---|
| M0 | 3.533 |
| M1 | 3.37 |
| M2 | 3.909 |
### Chart:
| Category | Met/Hcy |
|---|---|
| M0 | 24.799693993146008 |
| M1 | 13.511543408838245 |
| M2 | 36.56380198005434 |p = 0.0002
p = 0.026
F
E
### Chart: Methyl transfer from Ser
 to Met and H3K9me
| Category | Met | H3K9me1 | H3K9me3 |
|---|---|---|---|*
***
*
**
M0 M1 M2 M0 M1 M2
 20% O2 2% O2
3,3,2-2H-Ser
2H-Gly
SHMT2
T1C1CCA
MS
NO
T1C1CCA
B12
2H-Met
MAT
2H-SAM
2H-H3 K9me
