# Supplementary material for: Multi-OMICs analysis reveals metabolic and epigenetic changes associated with macrophage polarization
Source: J Biol Chem. 2022 Aug 27;298(10):102418. doi: 10.1016/j.jbc.2022.102418 (PMC9525912; doi:10.1016/j.jbc.2022.102418)
Supplement: Figure S6 [file mmc8.pptx]

## Slide 1
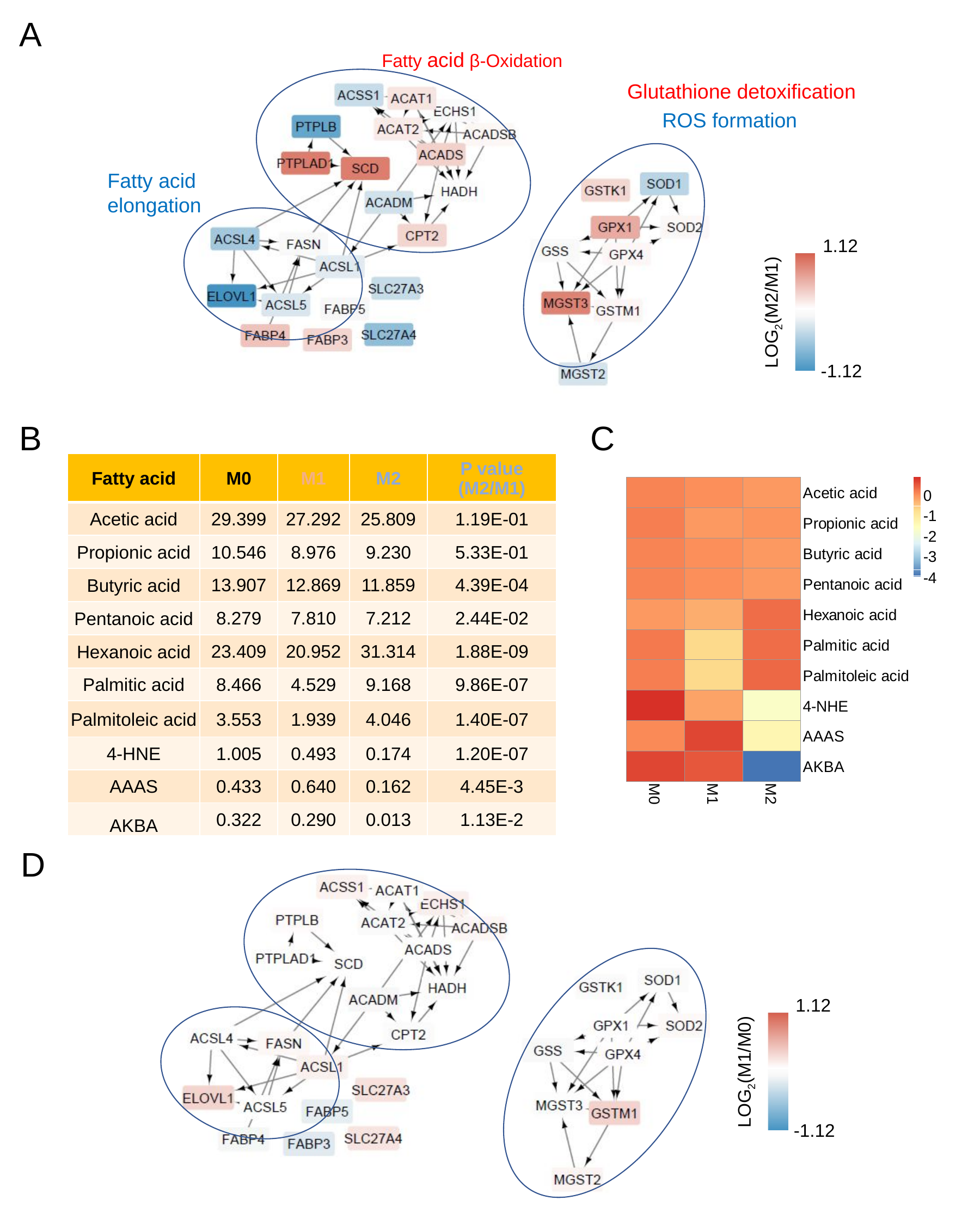

A
Fatty acid β-Oxidation
Glutathione detoxification
ROS formation
Fatty acid
elongation
1.12
LOG2(M2/M1)
-1.12
B
C
| Fatty acid | M0 | M1 | M2 | P value (M2/M1) |
| --- | --- | --- | --- | --- |
| Acetic acid | 29.399 | 27.292 | 25.809 | 1.19E-01 |
| Propionic acid | 10.546 | 8.976 | 9.230 | 5.33E-01 |
| Butyric acid | 13.907 | 12.869 | 11.859 | 4.39E-04 |
| Pentanoic acid | 8.279 | 7.810 | 7.212 | 2.44E-02 |
| Hexanoic acid | 23.409 | 20.952 | 31.314 | 1.88E-09 |
| Palmitic acid | 8.466 | 4.529 | 9.168 | 9.86E-07 |
| Palmitoleic acid | 3.553 | 1.939 | 4.046 | 1.40E-07 |
| 4-HNE | 1.005 | 0.493 | 0.174 | 1.20E-07 |
| AAAS | 0.433 | 0.640 | 0.162 | 4.45E-3 |
| AKBA | 0.322 | 0.290 | 0.013 | 1.13E-2 |
D
1.12
LOG2(M1/M0)
-1.12
